# Scalability of Schottky barrier metal-oxide-semiconductor transistors

**DOI:** 10.1186/s40580-016-0071-0

**Published:** 2016-05-16

**Authors:** Moongyu Jang

**Affiliations:** Department of Materials Science and Engineering, Hallym University, Chuncheon, Gangwon-do South Korea

**Keywords:** SB-MOSFETs, Schottky diode, Interface trap, Scaling

## Abstract

In this paper, the general characteristics and the scalability of Schottky barrier metal-oxide-semiconductor field effect transistors (SB-MOSFETs) are introduced and reviewed. The most important factors, i.e., interface-trap density, lifetime and Schottky barrier height of erbium-silicided Schottky diode are estimated using equivalent circuit method. The extracted interface trap density, lifetime and Schottky barrier height for hole are estimated as 1.5 × 10^13^ traps/cm^2^, 3.75 ms and 0.76 eV, respectively. The interface traps are efficiently cured by N_2_ annealing. Based on the diode characteristics, various sizes of erbium-silicided/platinum-silicided *n/p*-type SB-MOSFETs are manufactured and analyzed. The manufactured SB-MOSFETs show enhanced drain induced barrier lowering (DIBL) characteristics due to the existence of Schottky barrier between source and channel. DIBL and subthreshold swing characteristics are comparable with the ultimate scaling limit of double gate MOSFETs which shows the possible application of SB-MOSFETs in nanoscale regime.

## Introduction

Recently, semiconductor–metal junction-based electronic devices are being studied for the applications in nanometer regime as the alternative of conventional metal-oxide-semiconductor field-effect transistors (MOSFETs) [1–6]. In Schottky barrier MOSFETs (SB-MOSFETs), the source and drain are composed of silicide instead of impurity doped silicon. Thus the parasitic source and drain resistance can be efficiently eliminated and the process temperature can be reduced dramatically lower than 600 °C, giving the opportunity to use metal as gate electrode and high-K dielectric materials as gate insulator [1–4]. However, in SB-MOSFETs, silicon in channel region reacts with the deposited metals. This reaction can cause the generation of trap states, causing microscopic inhomogeneity of Schottky barrier height [7]. Thus, for the improvement of the device performance, the interface of Schottky diode should be carefully analyzed. Until now, current–voltage (*I*–*V*) measurement method has been widely used to explore the trap states in Schottky diode by evaluating diode ideality factor [7, 8]. However, there has been no established method on the quantitative evaluation of trap density in the Schottky diode, although it is well established in the metal-insulator-semiconductor system [9].

In SB-MOSFETs, most of the works are done in *p*-type transistors, using platinum-silicide because of its low Schottky barrier height (0.24 eV) for hole [6]. Although there are few works on *n*-type SB-MOSFETs, erbium-silicide is being considered as the promising candidate for the *n*-type SB-MOSFETs [5, 6]. However, erbium-silicide could generate heavy trap sites at silicon/erbium silicide interface. Thus, the study on the erbium-silicided Schottky diode characteristics, incorporating trap states with Schottky barrier height and their effects on the electrical characteristics of SB-MOSFETs are very important.

In this paper, the detailed characteristics of erbium-silicided Schottky diode, fabricated on the *p*-type silicon are introduced and the interface of Schottky diode is analyzed using the current–voltage and capacitance–voltage (*C*–*V*) measurement methods. Moreover, by incorporating equivalent circuit model with the *C*–*V* measurement method, trap density, lifetime and Schottky barrier height are extracted in erbium-silicided Schottky diode. Also, short channel characteristics of SB-MOSFETs are analyzed using drain induced barrier lowering (DIBL) and subthreshold swing (SS) characteristics. Also, the simple DIBL model of SB-MOSFETs is proposed and compared with the scaling theory of double gate (DG) MOSFETs.

## Experimental

### Fabrication of erbium-silicided Schottky diode

In conventional MOSFETs, Titanium, Cobalt and Nickel are widely being used for the silicidation process to minimize the parasitic resistance of impurity doped source and drain. But in this work, erbium is chosen as source and drain metal of *n*-type SB-MOSFETs, because of its low Schottky barrier height (0.28 eV) for electrons [5, 6]. The boron doped (100) *p*-type bulk silicon wafer is used for the erbium-silicided Schottky diode. The resistivity is 13.5–22.5 Ω cm and the corresponding doping concentration is about 1.0 × 10^15^ cm^−3^. After wafer cleaning, 100 nm thick SiO_2_ layer is grown on the wafers using thermal oxidation method at 1000 °C and the 250 × 250 μm region is opened using lithography and 30:1 BOE (buffered oxide etchant) etching. After erbium sputtering, erbium-silicide is formed by using rapid thermal annealing (RTA) technique. Annealing temperature and time and pressure are 500 °C, 5 min and 1.0 × 10^−6^ torr, respectively. The non reacted erbium is removed by using the mixture of H_2_SO_4_ and H_2_O_2_ (Sulfuric Peroxide mixture: SPM) SPM for 10 min. The volume ratio of H_2_SO_4_ and H_2_O_2_ is 1:1. The thickness of ErSi_1.7_ is about 55 nm which is confirmed by transmission electron microscopy. Platinum-silicide is formed on the boron heavily doped backside of *p*-type silicon wafers for ohmic contact for the accuracy of the measurements.

### Fabrication of SB-MOSFETs

**Fig. 1:**
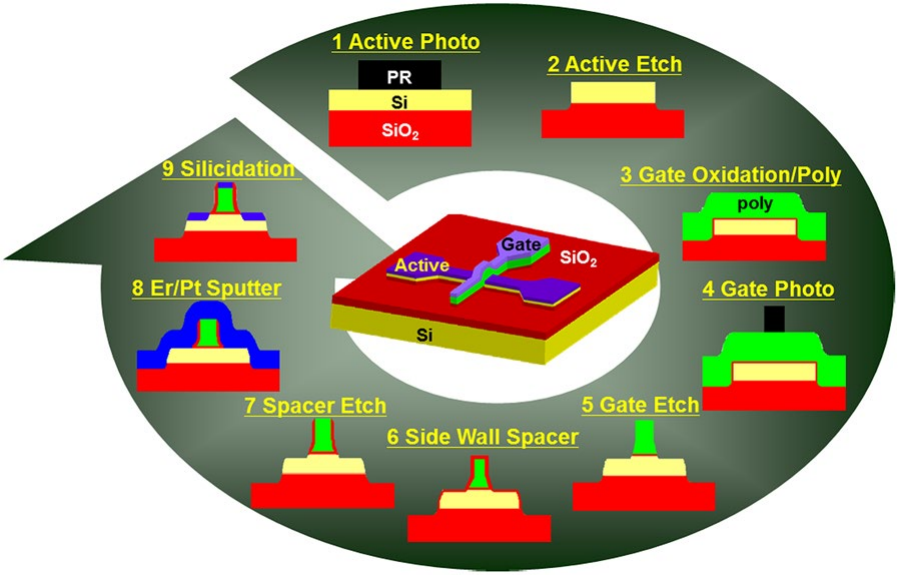
Schematic diagram of the fabrication process for Schottky barrier MOSFETs

As starting material, (100) *p*-type silicon-on-insulator (SOI) wafer is used. SOI wafer is boron doped with a resistivity of 13.5–22.5 Ω cm and the corresponding doping concentration is about 1.0 × 10^15^ cm^−3^. The thickness of the SOI and buried oxide (BOX) layer is 100 and 200 nm, respectively. The gate oxide is 5 nm thick SiO_2_, grown by thermal oxidation and the gate electrode is highly phosphorus doped *n*-type polycrystalline silicon. Electron-beam lithography is employed to define gate pattern. After gate etching, 30 nm thick gate sidewall spacer is formed by using thermal oxidation method. After blanket dry etching of gate sidewall spacer, 100 nm thick erbium and platinum are sputtered for *n*-type and *p*-type SB-MOSFETs, respectively. Erbium-silicide and platinum-silicide are formed by using rapid thermal annealing (RTA) technique. Annealing temperature and time is 500 °C and 5 min, respectively. The non-reacted erbium and platinum are removed by using SPM and aqua regia for 10 min, respectively. The formation of ErSi_1.7_ and PtSi phase are confirmed by x-ray diffraction (XRD) and Auger electron spectroscopy (AES) analysis. The sheet resistance are less than 30 and 10 Ω/sq for erbium-silicide and platinum-silicide, even if the line width is less than 100 nm. Thus, erbium and platinum are applicable in nanometer regime SB-MOSFETs manufacturing. The detailed process flow is summarized in Fig. 1.

## Results and discussion

### Analysis of erbium-silicided Schottky diode

**Fig. 2:**
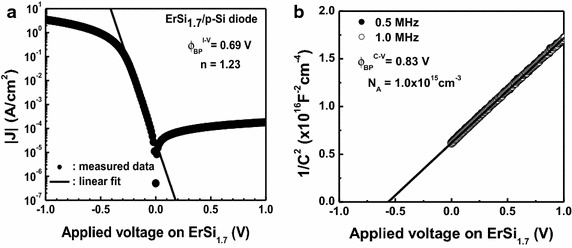
Room—temperature *I*–*V* and *C*–*V* curves of ErSi_1.7_/p-Si diode. The extracted Schottky barrier height for hole is 0.69 and 0.83 V from *I*–*V* (*Φ*_*BP*_^*I*–*V*^) and *C*–*V* (*Φ*_*BP*_^*C*–*V*^) method, respectively. In **a** and **b**, *n* and *N*_*A*_ denote ideality factor and doping concentration of substrate, respectively. Reprinted, with permission, from [10]

For the erbium-silicide/*p*-type silicon contact, Schottky barrier heights for hole are extracted as 0.69 and 0.83 eV from *I*–*V* (Fig. 2a) and *C*–*V* measurement (Fig. 2b), respectively [10]. The barrier heights determined by two methods give big difference, which causes the difficulty in the determination of Schottky barrier height. On the other hand, from the *I*–*V* measurement, the extracted ideality factor is 1.23 which implies the possible existence of traps within the depletion region of erbium-silicided Schottky diode. The existence of traps causes the microscopic inhomogeneity of Schottky barrier, which will in turn degrade the ideality factor, thus change the Schottky barrier height [7, 8]. Forwardly biased current transport at inhomogeneous Schottky barrier is dominated by low Schottky barrier patches, leading to the deduction of barrier height. Since, under usual circumstances, the *C*–*V* method yields an average Schottky barrier height for the whole diode [7]. To analyze the leakage current (*I*_*R*_) conduction mechanisms in reversely biased Schottky diode, Frenkel-Poole and Schottky emission models are considered [9, 11]. The plot of ln*(I*_*R*_ */E)* and ln*(I*_*R*_ */T* ^*2*^ *)* versus *E* ^*1/2*^ gives the linear curve for the Frenkel-Poole and Schottky emission and the slope can be expressed as fowllowing [11].

1 $$S = \frac{q}{nkT}\sqrt {\frac{q}{\pi \varepsilon }}$$

where, *n* = 1, 2 for the case of Frenkel-Poole and Schottky emission, respectively. Here, *q* is the electron charge quantity, *k* Boltzmann’s constant, *ε* permittivity, and *E* the maximum electric field in Schottky diode. The theoretically calculated slopes at 27 °C for Frenkel-Poole and Schottky emission is 0.0087 and 0.0043 (V/cm)^−1/2^, respectively.

**Fig. 3:**
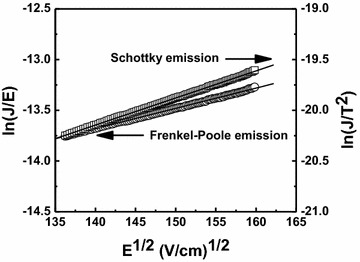
Electric field dependence of ErSi_1.7_/p-Si diode in the reverse bias region at 27 °C. The *circle* and *square symbol* correspond to the Frenkel-Poole and Schottky emission, respectively Reprinted, with permission, from [10]

Figure 3 shows ln*(I*_*R*_ */E)* and ln*(I*_*R*_ */T* ^*2*^ *)* versus *E* ^*1/2*^ curves obtained with the use of *I*–*V* data shown for the erbium-silicided Schottky diode in Fig. 2a. The slopes as determined from the fit to the data are extracted as 0.013 and 0.027 (V/cm)^−1/2^, for Frenkel-Poole and Schottky emission, respectively. The experimentally determined slope is slightly larger than the theoretical value for Frenkel-Poole case, but is much larger than the theoretical value for Schottky emission. Thus, the result is more consistent with Frenkel-Poole emission which implies the existence of deep trap level within depletion region of erbium-silicided Schottky diode.

**Fig. 4:**
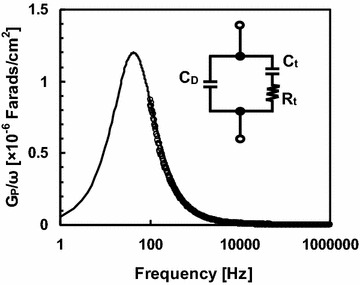
Frequency versus conductance loss plot. From this plot, the trap density (*D*_*t*_) and lifetime (*τ*) can be extracted. The reversely biased voltage is 0.2 V.* Inset* shows equivalent circuit of Schottky diode including trap states Reprinted, with permission, from [10]

The equivalent circuit of Schottky diode, including the trap states can be modeled as shown as inset of Fig. 4 [9]. In the figure, *C*_*D*_ *, C*_*t*_ and *R*_*t*_ are the semiconductor depletion-layer capacitance, traps associated capacitance and resistance, respectively. The product *C*_*t*_ *·R*_*t*_ is defined as the trap lifetime*τ*. The parallel branch of the equivalent circuit in Fig. 4 can be converted into a frequency-dependent *C*_*p*_ in parallel with a frequency-dependent conductance *G*_*p*_ and can be expressed as following [10].

2 $$\frac{{G_{p} }}{\omega } = \frac{{C_{t} \omega \tau }}{{1 + \omega^{2} \tau^{2} }},\quad C_{p} = C_{D} + \frac{{C_{t} }}{{1 + \omega^{2} \tau^{2} }}$$

where, *ω* is the angular frequency.

In (2), the plot of *G*_*p*_ */ω* versus *ωτ* goes through maximum when *ωτ* = 1, and gives *τ* directly. The value of *G*_*p*_ */ω* at the maximum is *C*_*t*_/2. Thus, equivalent parallel conductance, divided by angular frequency gives *C*_*t*_ and *τ* directly from the measured conductance. The trap density is obtained by using the relation *D*_*t*_ = *C*_*t*_ */qA*, where *A* is the diode area. By using the extracted value *C*_*t*_ in *G*_*p*_ */ω* relation, *C*_*D*_ can be extracted directly by using *C*_*p*_. Also, *C*_*t*_ and*τ* can be evaluated by using *C*_*p*_ relation. But in this case, inaccuracy of extracted values can exist because of the sensitive dependence of *C*_*p*_ to measured frequency, *ω.* For the consideration of external resistance including substrate and contact resistance, serial connection of additional resistor can be added.

Figure 4 shows the plot of *G*_*p*_ */ω* versus frequency with the 0.2 V reverse bias condition. The conductance and capacitance are measured using HP4285A impedance analyzer. In figure, the circle and solid line represent measured and fitted data, respectively. From the curve fitting, the extracted *C*_*t*_, *D*_*t*_ and *τ* value are 2.4 × 10^−6^ Farads/cm^2^, 1.5 × 10^13^ traps/cm^2^ and 3.75 ms, respectively. The extracted *C*_*D*_ value is 10.6 × 10^−9^ Farads/cm^2^. The extracted interface-trap density is higher compared with the typical values in SiO_2_ interface [12]. This interface-trap can be cured using hydrogen annealing.

**Fig. 5:**
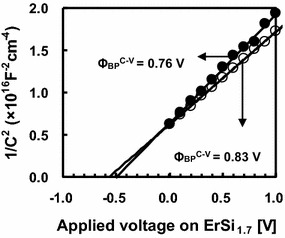
Re-extraction of Schottky barrier height by eliminating the capacitance associated with trap Reprinted, with permission, from [10]

By using the aforementioned method, depletion capacitance (*C*_*D*_) values are extracted for the various reverse bias conditions. Figure 4 shows the plot of 1/*C*_*D*_^2^ versus reverse voltage and open and closed circles represent the as-measured (*C*_*P*_) and corrected depletion capacitance (*C*_*D*_) data, respectively. *C*_*P*_ includes *C*_*t*_, giving the wrong Schottky barrier height in the plot of 1/*C* ^2^ versus reverse voltage. The slope of corrected data has higher values than that of as-measured data because *C*_*t*_ is eliminated from *C*_*P*_. From Fig. 5, the re-extracted Schottky barrier height is 0.76 eV for hole and 0.36 eV for electron. The extracted value is consistent with reported photoemission method, which shows the validity of newly developed method [13]. The extracted Schottky barrier height of erbium-silicide is higher compared with the value extracted in SB-MOSFETs results (0.28 eV) [3]. In SB-MOSFETs, image force induced Schottky barrier height lowering is severe due to the electrically induced inversion carriers. At strong inversion condition, the estimated barrier lowering value is almost 0.1 eV [5]. Thus, in SB-MOSFETs, the effective Schottky barrier height can be lower than the value extracted by Schottky diode measurement. Also, when interface traps are considered in Schottky interface, Fermi level pinning effect should be analyzed. The existence of trap states cause the strong pinning of Fermi level of being considered silicide material. For example, the expected Fermi level of erbium is around −3.2 eV which means the zero-Schottky barrier for electron. However, the electrically extracted barrier height of erbium-silicide is around 0.28 eV for electron. Thus, Fermi level pinning and trap state should be carefully controlled. More deep level discussions will be made in another report.

### Scalability of SB-MOSFETs

**Fig. 6:**
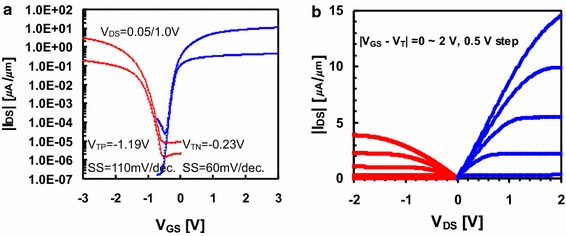
I_DS_-V_GS_ (**a**) and I_DS_-V_DS_ (**b**) characteristics of 20 μm long channel *n/p*-type SB-MOSFET Reprinted, with permission, from [15]

Figure 6 shows I_DS_-V_GS_ characteristics of the 20 μm long channel *n/p*-type SB-MOSFETs. The gate oxide and spacer thickness is 5 and 15 nm, respectively. Both the *n/p*-type SB-MOSFETs show high on/off current ratio, larger than I_on_/I_off_ > 10^5^ with low leakage current (I_LKG_ < 100 pA/μm). The on/off ratio and the leakage current level is the highest and lowest values compared with previously published data in *n*-type SB-MOSFETs [6]. Also, DIBL is almost suppressed in both *n/p*-type SB-MOSFETs and the SS value is 60 mV/decade in *n*-type SB-MOSFETs.

**Fig. 7:**
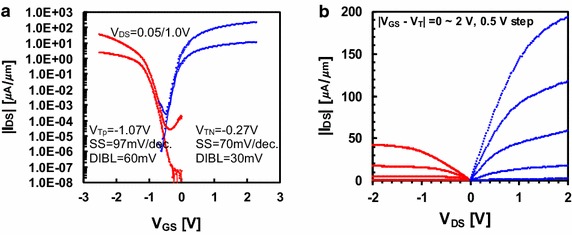
I_DS_-V_GS_ (**a**) and I_DS_-V_DS_ (**b**) characteristics of 100 nm gate length *n/p*-type SB-MOSFET Reprinted, with permission, from [15]

Figure 7 shows the I_DS_-V_GS_ characteristics of erbium-silicided 100 nm gate length *n/p*-type SB-MOSFETs. Also, this 100 nm gate length *n/p*-type SB-MOSFET shows excellent short channel characteristics. The measured SS and DIBL values are 70 mV/decade and 30 mV, respectively, for *n*-type SB-MOSFETs.

**Fig. 8:**
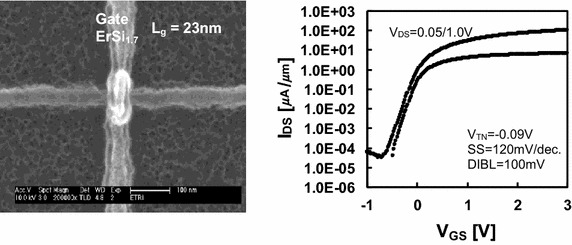
SEM image and I_DS_-V_GS_ characteristics of 23 nm *n*-type SB-MOSFET Reprinted, with permission, from [15]

Figure 8 shows the SEM image and electrical I_DS_-V_GS_ characteristics of 23 nm gate length *n*-type SB-MOSFETs [15]. Although the substrate boron doping concentration is 10^15^ cm^−3^, short channel effect is sufficiently suppressed due to the existence of Schottky barrier between source and channel. The existence of interface traps can severely affect the short channel characteristics in SB-MOSFETs, especially for the low doped substrate case due to the severe penetration of drain field into the source/channel interface. The penetration of drain field can cause the interface trap mediated leakage current, giving the degradation of SS value and leakage current characteristics.

**Fig. 9:**
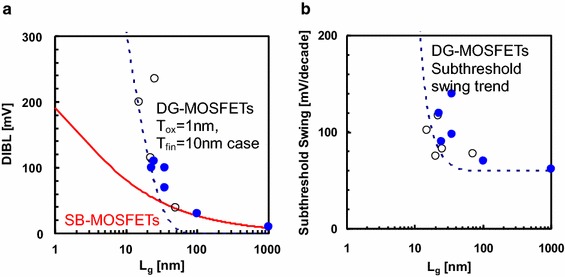
DIBL (**a**) and subthreshold swing (**b**) characteristics of SB-MOSFETs

**Fig. 10:**
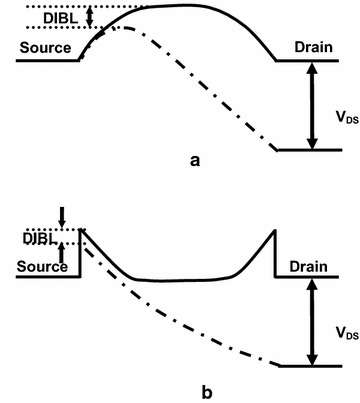
Different DIBL mechanisms in MOSFETs (**a**) and SB-MOSFETs (**b**)

Figure 9 shows the DIBL (a) and SS (b) characteristics of SB-MOSFETs with the variation of gate length. In Fig. 8a, solid and dotted line represents theoretical DIBL characteristics of SB-MOSFETs and DG-MOSFETs, respectively and the open circles are plotted from the published data [6] and the closed circles are the data from this work. The scaling theory of DG-MOSFETs can be found in [14]. In the calculations of DIBL in DG-MOSFETs, gate oxide and body thickness are assumed as 1 and 10 nm, respectively. Note that these assumed values correspond to the ultimate minimum values in device technology. The DIBL characteristics of SB-MOSFETs are better than DG-MOSFETs. The reason for this is due to the existence of the Schottky barrier between source and channel. This is explained in Fig. 10. In DG-MOSFETs, the subthreshold characteristics, including DIBL and SS, are mainly determined by the built-in potential. In short channel device, as the drain voltage increases, built-in potential between source and channel decreases, giving DIBL effect. But in SB-MOSFETs, the subthreshold characteristics are mainly determined by the Schottky barrier. Thus DIBL characteristics of SB-MOSFETs can be described by the Schottky barrier lowering due to the drain voltage. In SB-MOSFETs, the decrease of threshold voltage with the increase of drain voltage, due to the Schottky barrier lowering, can be expressed as following.

3 $$\Updelta {\text{V}}_{\text{T}} = \sqrt {\frac{q}{4\pi \varepsilon }} \left[ {\sqrt {\frac{{V_{DS}^{H} }}{{L_{eff} }}} - \sqrt {\frac{{V_{DS}^{L} }}{{L_{eff} }}} } \right]$$

where, *L*_*eff*_ means the effective gate length and *V*_*DS*_^*H*^ and *V*_*DS*_^*L*^ means high and low drain voltage, respectively.

In Fig. 9b, dotted line represents theoretical SS characteristics of DG-MOSFETs, with 1 nm gate oxide and 10 nm body thickness. As shown, the SS characteristics of SB-MOSFETs are almost compatible with ultimately scaled DG-MOSFETs. However there exist deviations of DIBL values of SB-MOSFETs from the theoretical prediction. The reason for these deviations are due to the interface trap states between silicon and silicide interface. As analyzed in Fig. 3, most of the trap states contribute to Frenkel-Poole emission which causes the degradation of DIBL and also SS characteristics in SB-MOSFETs. Thus, the control of the interface trap states in SB-MOSFETs are the important key factor for the improvement of DIBL and SS characteristics. One efficient method for the reduction of interface traps is N_2_ annealing and the detailed method and the results are reported in [15].

## Conclusion

Erbium-silicided Schottky diode is fabricated on the *p*-type silicon and the electrical characteristics is analyzed using the *I*–*V* and *C*–*V* measurement methods. From *I*–*V* analysis, the major leakage current conduction mechanism of reversely biased Schottky diode is due to the Frenkel-Poole emission originating from the existence of deep trap level in the depletion region of erbium-silicided Schottky diode. The trap density and lifetime are evaluated using equivalent circuit modeling method and the extracted trap density and lifetime are 1.5 × 10^13^ traps/cm^2^ and 3.75 ms, respectively. The corrected Schottky barrier height (0.76 eV) is extracted by eliminating the parallel connected capacitance associated with trap using equivalent circuit modeling method. Also, SB-MOSFETs are manufactured and the electrical characteristics are analyzed. In SB-MOSFETs, DIBL is strongly suppressed due to the existence of Schottky barrier between source and channel. DIBL and SS characteristics of SB-MOSFETs are compatible with the ultimately scaled DG-MOSFETs which shows the possible application of SB-MOSFETs in nanoscale regime as the alternative to the MOSFETs.
